# Outcomes and Prognostic Factors in Patients With Untreated Head and Neck Squamous Cell Carcinoma

**DOI:** 10.7759/cureus.77141

**Published:** 2025-01-08

**Authors:** Hisashi Kessoku, Yosuke Mizunari

**Affiliations:** 1 Department of Otorhinolaryngology, Jikei University Kashiwa Hospital, Kashiwa, JPN

**Keywords:** best supportive care, head and neck cancer, natural history, social isolation, squamous cell carcinoma, untreated

## Abstract

Objectives:Although several studies have reported the treatment prognosis in squamous cell carcinoma of the head and neck, few studies exist on the prognosis and mortality-related risk factors in untreated cases. This study aimed to determine the outcomes of patients with head and neck squamous cell carcinoma who underwent no treatment and investigate the associated factors.

Methods:This retrospective, single-institution study initially included 718 patients with head and neck cancer who visited our hospital between January 2015 and December 2021; 43 untreated patients were included in the final analysis. This study was conducted in Japan, where universal health insurance ensures access to treatment for all citizens.

Results:The median patient age was 79 years, with a predominance of male patients. The primary reasons for patients being untreated were patient refusal, dementia, and advanced age. The median survival time for untreated patients was 20.5 weeks, with 90% succumbing within one year of their first visit. Univariate analyses showed that an advanced clinical stage (stage IV) was a major determinant of survival. In patients with stage IV disease, living alone or without family support was the sole significant predictor of a poor prognosis.

Conclusions: Our results inform healthcare providers about the consequences of not providing treatment and patients regarding treatment choices.

## Introduction

Head and neck cancer is a general term used for cancers arising in the head and neck region in various subpopulations. Approximately 90% of cancers originate from the oral cavity, pharynx, or larynx; most of these are squamous cell carcinomas [1]. The median five-year survival rate is only 60%, although curable patients are treated with surgery, radiotherapy, chemoradiotherapy alone, or in combination, in a multidisciplinary fashion [2]. Additionally, palliative treatments, such as chemotherapy, are used for patients with distant metastases that cannot be cured; however, the median survival is even worse at 15 months [3]. Moreover, some patients refuse or are deemed ineligible for treatment, necessitating the provision of additional support and appropriate resource allocation to ensure their proper management [4].

However, existing studies on untreated head and neck squamous cell carcinoma outcomes are limited and reveal variations across countries due to differences in major ethnic groups, health insurance, or care system accessibility, underscoring the necessity for context-specific research tailored to diverse healthcare environments [4-8]. Japan, in particular, has a universal health insurance system that requires all citizens to enroll in public health insurance programs. This system allows individuals to access necessary medical care with minimal financial burden and promotes a high standard of healthcare through mutual support among insured individuals. In addition to offering access to high-cost medical care, Japan's healthcare system contributes to the country's long life expectancy and one of the most favorable medical environments in the world. These factors are critical in understanding healthcare decisions and treatment outcomes in Japan, including the reasons why some patients with head and neck cancer remain untreated. Therefore, this study aimed to determine the outcomes of patients with head and neck squamous cell carcinoma who did not undergo any treatment and investigate the associated factors, exploring the implications of non-intervention and identifying potential strategies to better support and manage patients who refuse to undergo treatment.

## Materials and methods

Ethics statements

This study was approved by the Institutional Review Board of the University of Jikei (approval number: 35-014(11635)) and was conducted in accordance with the Helsinki Declaration of 1975, as revised in 2013. Participants were provided information about the research without obtaining individual informed consent and an opportunity to refuse participation (opt-out). To protect patient confidentiality, all patient details were de-identified, ensuring that no individual could be identified in any way.

Study design and patient involvement

In this retrospective study, we conducted an analysis of patients with head and neck cancer who visited the Department of Otorhinolaryngology at the Jikei University Kashiwa Hospital, a tertiary medical center located in Chiba, Japan, between January 2015 and December 2021. Patients were selected consecutively for inclusion, ensuring that all eligible patients meeting the inclusion criteria during the study period were analyzed. This study adheres to the Strengthening the Reporting of Observational Studies in Epidemiology (STROBE) guidelines [9]. The inclusion criteria focused on patients with untreated squamous cell carcinoma originating from the mucosa of the oral cavity, pharynx, or larynx. In this study, "untreated" was defined as patients who had not undergone any definitive therapy for the tumor, such as surgery, radiation therapy, or chemotherapy. However, patients who received supportive care measures, including tracheostomy, percutaneous endoscopic gastrostomy (PEG) insertion, or central venous (CV) port placement, were included in the analysis. For patients with early-stage disease, appropriate treatment options, such as surgery or radiation therapy, were discussed and proposed during clinical consultations. Ultimately, the decision to forgo these treatments was made by the patients themselves, based on their preferences or personal circumstances.

Patients were excluded if they had head and neck cancer other than squamous cell carcinoma, had received radical or palliative treatments such as chemotherapy or palliative radiotherapy, or had squamous cell carcinoma originating from non-mucosal sites, such as the skin of the ear canal or the salivary glands. Additionally, patients with carcinoma of unknown primary origin or squamous cell carcinoma of the primary mucosa who had not undergone treatment but had a poor prognosis due to malignant diseases outside the head and neck region, such as advanced leukemia, metastatic colorectal cancer, or metastatic lung cancer, were also excluded.

**Figure 1:**
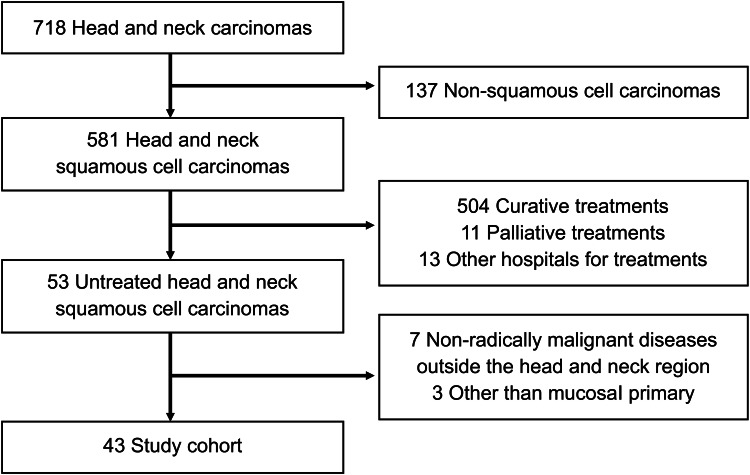
Flow diagram of study cohort selection

After applying the inclusion and exclusion criteria, data collection was performed to analyze the eligible patients. A total of 718 patients were initially identified. Among these, 137 had non-squamous cell carcinoma, and 504 patients underwent curative treatment. Additionally, 11 patients received palliative treatment, and 12 were referred to other hospitals for treatment. After these exclusions, 53 patients with untreated squamous cell carcinoma of the head and neck remained eligible for further evaluation. Following a review, 10 patients were excluded due to non-mucosal primary sites or comorbid malignant conditions, leaving 43 patients for the final analysis (Figure 1). All patient information was obtained from medical records, including sex, age, Eastern Cooperative Oncology Group Performance Status (ECOG PS) [10], tumor site, and tumor stage (for cases before 2018, the stage was reassessed according to the Union for International Cancer Control TNM classification, 8th edition). Information on PEG or CV port, tracheotomy, processes that were refused, follow-up information, and social isolation (defined as living alone or having family members who lived far away or were unwilling to take care of the patient) was extracted and analyzed. Survival was defined as the period from the first visit to the date of death from any cause. The date of death could be identified for all patients.

Statistical analysis

Fisher's exact test was utilized for binary and ordinal variables to analyze patient characteristics, while the Mann-Whitney U test was employed for continuous variables. Survival analysis was performed using the Kaplan-Meier model, and the Cox proportional hazards model was used to compare the survival between the strata of each variable. All statistical analyses were performed using Stata Statistical Software: Release 17 (2021; StataCorp LLC, College Station, Texas, United States). P<0.05 was considered significant.

## Results

Patients

Among the 43 patients with untreated head and neck squamous cell carcinoma, the median age was 79 years (interquartile range: 74-85 years). There were 36 male and seven female patients. The ECOG PS was 0-2 in 31 patients and 3-4 in 12 patients. The primary tumor sites were the oral cavity (three patients), larynx (eight patients), oropharynx (12 patients), hypopharynx (18 patients), and other sites (maxillary sinus and nasopharynx patients). With 39 patients in stages III and IV, more than 90% of the patients were at advanced stages of the disease. Tracheostomies were performed in 12 patients, often performed urgently at the first visit, given the risk of airway obstruction due to the tumor. In seven cases, PEGs and CV ports were added to enable discharge to home or transfer to a facility for patients with oral intake difficulties. In total, 28 patients lived alone or had family members who could not care for them (Table 1).

**Table 1:** General characteristics of the participants Data are presented as unweighted number (percentage) of patients unless otherwise indicated. ECOG: Eastern Cooperative Oncology Group; PS: Performance Status; PEG: percutaneous endoscopic gastrostomy; CV: central venous

Variable	Overall n=43
Age (year), median (interquartile range)	79 (74-85)
Male sex	36 (83)
ECOG PS	
0-2	31 (72)
3-4	12 (28)
Site	
Oral cavity	3 (7)
Oropharynx	12 (28)
Hypopharynx	18 (42)
Larynx	8 (18)
Others (maxillary sinus, nasopharynx)	2 (5)
Clinical stage	
Stage I	3 (7)
Stage II	1 (2)
Stage III	9 (21)
Stage IV	30 (70)
Tracheostomy	12 (28)
PEG tube or CV port	7 (16)
Social isolation: Live alone or no family members available for care	28 (65)

The reasons for not receiving treatment were a single factor or combination of several factors, with patient refusal being the most common (20 patients), followed by dementia or delirium (11 patients) and advanced age (10 patients), which may reflect underlying concerns about physical health and the perceived ability to tolerate cancer treatment. Five patients went untreated because of fatal events related to the tumor before treatment (two patients had a stroke due to Trousseau syndrome, two patients had asphyxia due to rapid tumor growth, and one patient had tumor hemorrhage) (Table 2).

**Table 2:** Reason for no treatment Unless otherwise indicated, data are presented as unweighted number (percentage) of patients. PS: Performance Status

Reason	Overall n=43
Patient refusal	20 (46)
Advanced age	10 (23)
Poor PS	7 (16)
Dementia/delirium	11 (25)
Many comorbidities	2 (5)
Pre-treatment tumor-related fatal events	5 (12)

Survival

**Figure 2:**
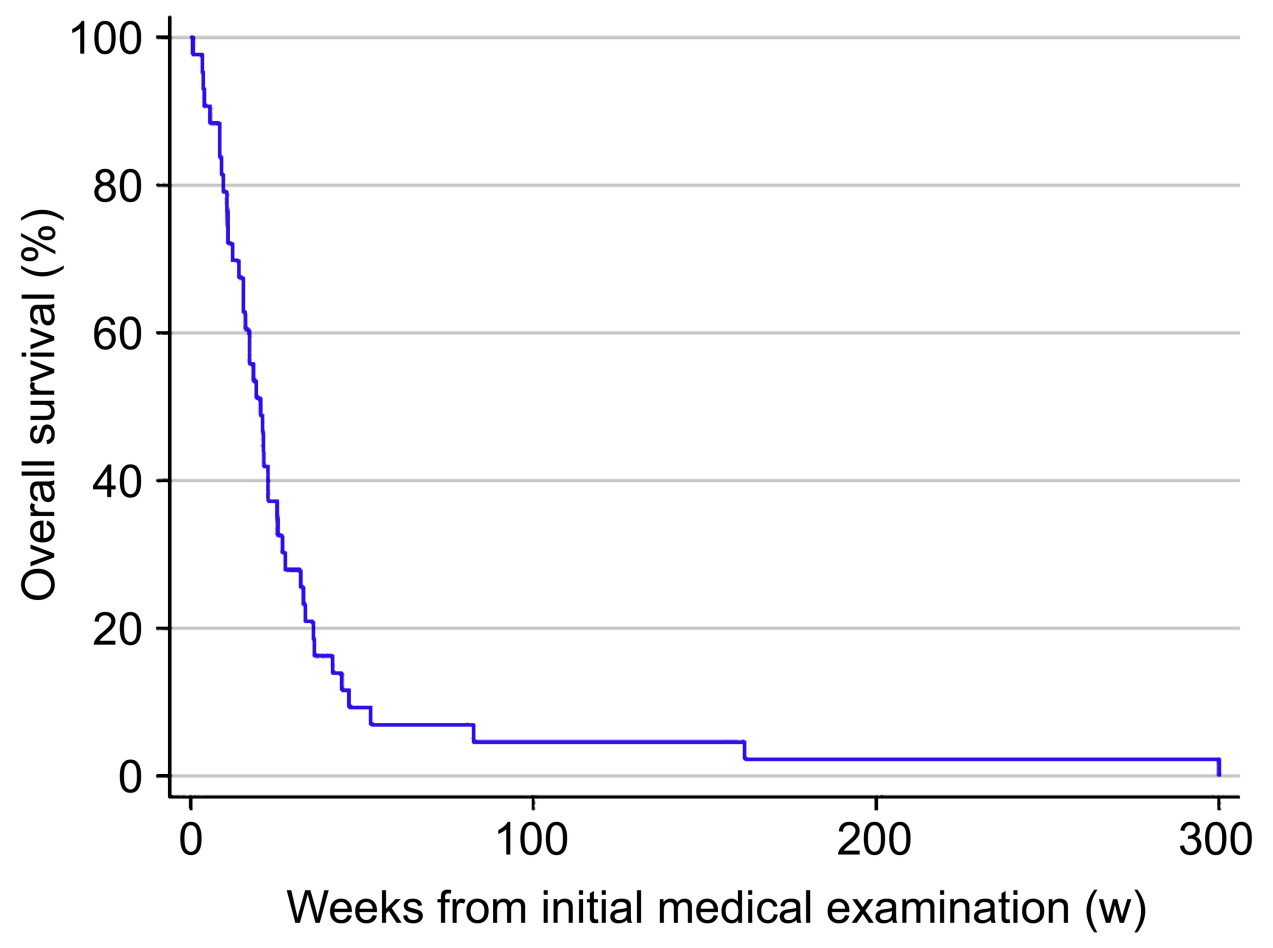
Kaplan-Meier analysis of overall survival for untreated patients with head and neck squamous cell carcinoma

The median survival of untreated patients was 20.5 weeks, and approximately 90% of patients died within one year of the initial diagnosis. The survival rates at six months and one year were 30.2% and 7% (95% confidence interval (CI): 17.4-44.1% and 1.8-17.1%), respectively (Figure 2).

Determinants of survival

Except for the advanced clinical stage (stage IV (hazard ratio (HR)=2.24; 95% CI: 1.11-4.52; p=0.024)), none of the patient factors were associated with overall survival (OS) in univariate analyses (Table 3). Therefore, we performed separate subgroup analyses for stage IV and other clinical stages (Table 4). Both poor ECOG PS (HR=2.60; 95% CI: 1.14-5.96; p=0.024) and social isolation (HR=3.82; 95% CI: 1.45-10.09; p=0.007) were poor prognostic factors in the stage IV subgroup in univariate analysis. A multivariate analysis for stage IV patients was performed using ECOG PS and social isolation, the two significant factors identified in the univariate analysis. This analysis revealed that social isolation (HR=3.13; 95% CI: 1.09-8.93; p=0.033) was the sole significant poor prognostic factor (Table 5).

**Table 3:** Univariate analysis of the overall study cohort ECOG: Eastern Cooperative Oncology Group; PS: Performance Status; PEG: percutaneous endoscopic gastrostomy; CV: central venous; CI: confidence interval

	Univariate analysis		
	Hazard ratio	95% CI	P-value
Age	0.99	0.95-1.03	0.49
Male sex	1.18	0.52-2.67	0.69
ECOG PS			
0-2	Reference	-	-
3-4	1.38	0.70-2.70	0.36
Tumor site			
Oral cavity	2.48	0.72-8.52	0.15
Oropharynx	0.94	0.47-1.91	0.87
Hypopharynx	1.41	0.75-2.68	0.29
Larynx	0.54	0.25-1.19	0.13
Others (maxillary sinus, nasopharynx)	1.06	0.25-4.43	0.94
Clinical stage			
Stage I	0.26	0.06-1.10	0.066
Stage II	1.35	0.18-10.02	0.77
Stage III	0.68	0.32-1.42	0.30
Stage IV	2.24	1.11-4.52	0.024
Tracheostomy	0.93	0.47-1.83	0.84
PEG tube or CV port	0.8	0.35-1.82	0.59
Social isolation	1.54	0.80-2.96	0.20

**Table 4:** Comparisons of characteristics between patients with stage I-III and stage IV disease *: Mann-Whitney U test; †: Fisher's exact test Data are presented as unweighted number (percentage) of patients unless otherwise indicated. ECOG: Eastern Cooperative Oncology Group; PS: Performance Status; PEG: percutaneous endoscopic gastrostomy; CV: central venous

Variable	Stage I-III (n=13)	Stage IV (n=30)	P-value
Age (year), median (interquartile range)	79 (76-84)	80 (74-85)	0.95*
Male sex	10 (77)	26 (87)	0.66†
ECOG PS			
0-2	10 (77)	21 (70)	0.73†
3-4	3 (23)	9 (30)	
Site			
Oral cavity	0 (0)	3 (10)	0.22†
Oropharynx	5 (38)	7 (23)	
Hypopharynx	3 (23)	15 (50)	
Larynx	4 (31)	4 (13)	
Others (maxillary sinus, nasopharynx)	1 (8)	1 (3)	
Tracheostomy	4 (31)	8 (27)	1.00†
PEG tube or CV port	4 (31)	3 (10)	0.17†
Social isolation	9 (69)	19 (63)	1.00†

**Table 5:** Univariate and multivariate analysis by subgroup ECOG: Eastern Cooperative Oncology Group; PS: Performance Status; PEG: percutaneous endoscopic gastrostomy; CV: central venous; CI: confidence interval

	Univariate analysis						Multivariate analysis		
	Stage I-III			Stage IV					
	Hazard ratio	95% CI	P-value	Hazard ratio	95% CI	P-value	Hazard ratio	95% CI	P-value
Age	1.02	0.95-1.11	0.54	0.95	0.90-1.00	0.055	-	-	-
Male sex	0.60	0.15-2.41	0.47	1.82	0.54-6.14	0.33	-	-	-
ECOG PS									
0-2	Reference	-	-	Reference	-	-	Reference	-	-
3-4	0.72	0.19-2.71	0.62	2.60	1.14-5.96	0.024	1.58	0.63-3.92	0.33
Site									
Oral cavity	-	-	-	1.69	0.49-5.84	0.41	-	-	-
Oropharynx	1.17	0.34-3.96	0.80	0.98	0.39-2.45	0.96	-	-	-
Hypopharynx	1.29	0.33-5.03	0.71	1.12	0.53-2.38	0.77	-	-	-
Larynx	0.74	0.22-2.53	0.63	0.53	0.18-1.56	0.25	-	-	-
Others (maxillary sinus, nasopharynx)	0.90	0.11-7.29	0.92	6.74	0.75-60.28	0.088	-	-	-
Tracheostomy	0.66	0.20-2.26	0.51	1.23	0.53-2.82	0.63	-	-	-
PEG tube or CV port	0.75	0.22-2.53	0.65	2.33	0.68-8.01	0.18	-	-	-
Social isolation	0.84	0.24-2.91	0.79	3.82	1.45-10.09	0.007	3.13	1.09-8.93	0.033

## Discussion

In this study, we determined the outcome of 43 patients with untreated squamous cell carcinoma of the head and neck and confirmed that stage IV clinical stage was a poor prognostic factor in the univariate analysis. We also found that social isolation (living alone or without family support) was the sole significant poor prognostic factor in patients with stage IV disease. Known factors contributing to the lack of treatment include age [4,7,8], race [4,11], obesity [8], unmarried status [4], economic reasons (such as a lack of private insurance or low income) [4,7], poor clinical status [5,8], unresectable advanced tumors [5], tumor location (oral, pharyngeal site) [7,11], advanced disease stage [11], multiple distant metastases [6], serious comorbidities [6], and patient refusal [5,6]. The factors contributing to a lack of treatment in this study were patient preference (patient refusal), advanced age (which may reflect underlying concerns about physical health and the perceived ability to withstand treatment), presence of dementia or delirium, poor ECOG PS, comorbidities, fatal events related to pre-treatment tumors, and a combination of these factors. There were no cases of non-treatment for racial or economic reasons, which differs from previous studies. This may be because Japan is not a multi-ethnic country and has a universal health insurance system, making it relatively easy to receive treatment. Notably, five patients were left untreated owing to pre-treatment tumor-related fatal events (cerebral infarction due to Trousseau syndrome, asphyxia due to rapid tumor growth, and tumor hemorrhage). Curative treatment was a feasible option for four of these five cases. As 504 patients were treated curatively at our hospital, approximately 1% of patients will go untreated owing to these fatal events, even if curative treatment is planned.

Previous studies in other regions have shown that untreated patients with head and neck squamous cell carcinoma account for about 10% of all patients and their prognosis is poor, with a median OS of 11.5 weeks to 12 months [4-8,11]. The median OS in our study was 20.5 weeks, similar to the results of previous studies. However, some patients classified as stage I have survived for over five years, even if they were untreated [11]. The only patient in this study who survived for over five years had stage I oropharyngeal cancer.

Reports on poor prognostic factors in patients with untreated squamous cell carcinoma of the head and neck have included advanced T-factors [8], advanced clinical stage [5], hypopharyngeal cancer [4], and poor performance status [5,11]. Advanced clinical stage (stage IV) was a poor prognostic factor in the present study. In addition, although limited to patients in stage IV, social isolation (living alone or having family members who lived far away or lacked the motivation to care for the patient) was newly found to be a poor prognostic factor.

Social isolation has been associated with increased mortality in patients without cancer [12] and poor prognosis in older patients with head and neck cancer [13]. Previous studies have shown that spouses and partners play a critical role in encouraging timely medical consultations, which is why married individuals are often diagnosed at earlier stages of the disease [14,15]. Moreover, spouses and partners influence treatment decisions, encouraging patients to pursue more aggressive therapeutic options [16]. They also provide emotional support [17], promote healthy behaviors, and strengthen caregiving efforts [18]. In contrast, socially isolated patients are more likely to delay seeking medical care, leading to more advanced disease at diagnosis, and may be less likely to undergo curative or definitive treatments even when these options are available. While patients with less advanced stages of head and neck cancer may survive with smaller support networks, the results of this study suggest that social isolation may contribute to poor outcomes in patients with advanced untreated head and neck cancer. Social support significantly contributes to patient activation, fostering greater engagement in treatment and adherence to prescribed regimens. This effect is particularly beneficial for older adults and vulnerable individuals, enhancing their ability to cope with cancer throughout the treatment process [19]. These findings underscore the importance of strategies to strengthen social support systems, which could improve treatment acceptance and outcomes in this patient group.

This study had some limitations. The findings of this study should be interpreted with caution due to its retrospective design and small sample size, which limited the ability to perform comprehensive multivariate analyses for all patients. Although we identified social isolation as a significant poor prognostic factor in stage IV patients, further validation through large-scale prospective studies is necessary to confirm its role. Additionally, missing or incomplete data from medical records may have affected the results. Finally, this was a single-center study. Nationwide data collection and multi-center studies are needed to provide a more accurate understanding of the natural history of untreated head and neck squamous cell carcinoma in Japan.

## Conclusions

The prognosis for untreated head and neck squamous cell carcinoma within this study cohort was notably poor, with 90% of patients dying within one year. Social isolation was suggested to be associated with worse survival outcomes, particularly in patients with stage IV disease. Despite the limitations acknowledged in this research, our findings mark a significant contribution to the field. This study is the first to document the outcomes of patients with untreated head and neck squamous cell carcinoma in Japan, laying the groundwork for a deeper understanding of its natural history and associated factors. The insights gained are indispensable for advancing patient management and designing interventions tailored to improve outcomes for this specific patient group. We believe that the results of this study will not only assist healthcare providers and patients in making more informed treatment decisions but also pave the way for enhanced care strategies for untreated patients with advanced stage IV cancer.
